# Voices from Health Care Providers: Assessing the Impact of the Indian Assisted Reproductive Technology (Regulation) Act, 2021 on the Practice of IVF in India

**DOI:** 10.1007/s13224-023-01815-2

**Published:** 2023-09-01

**Authors:** Jaydeep Tank, Prabha Kotiswaran, Parikshit Tank, Dev Tank, Jash Tank

**Affiliations:** 1 Ashwini Maternity and Surgical Hospital, Mumbai, India; 2 Dickson Poon School of Law, King’s College London, London, UK; 3 Jindal Global Law School, Mumbai, India; 4 DY Patil University School of Medicine Navi Mumbai, Mumbai, India

**Keywords:** ART Act, ART, Survey, Donors, IVF, Doctors, Surrogacy, Gametes

## Abstract

The regulatory vacuum in the field of ART in India was filled when in December 2021, the Assisted Reproductive Technology (Regulation) Act, 2021 (ART Act) (https://egazette.nic.in/WriteReadData/2021/232025.pdf) and the Surrogacy (Regulation) Act, 2021 (SR Act) were passed. We surveyed medical professionals to understand their knowledge, attitude and perception towards the Acts and to offer an initial, snapshot assessment of their impact on the medical community. The government has already signalled its intent to implement the Acts and has published several notifications/gazettes to clarify and amend the issues surrounding the Acts (https://artsurrogacy.gov.in/NationalArtSurrogacy/faces/HomePage.xhtml#). We hope that these responses will help to voice the thoughts, concerns and suggestions from of ART service providers for ART to further clarify and rationalise the laws. Infertility is already a much stigmatised problem which deserves to be a higher public health priority. While the laws are a welcome step, changes in both laws is are the need of the hour to make ART more accessible, available and affordable to the millions of couples who need these services and for the health care providers who to be able to deliver them.

## Introduction

Assisted Reproductive Technologies (ARTs) have played a crucial role in helping individuals and couples who experience infertility to form families. This often involves third parties such as gamete donors and surrogates. Even now, a large portion of the estimated 27 million infertile Indian couples, especially the poor lack easy access to ARTs. The provision of ART in India occurs overwhelmingly via the private sector. Discussions for regulating the ART services go back over twelve years and more.

The Indian government has explored almost every possible legal approach to ARTs and surrogacy [1]. Sixteen years, eight drafts, and three parliamentary committees later, the legislative process finally concluded with the Parliament’s passage in December 2021 of the Assisted Reproductive Technology (Regulation) Act, 2021 (ART Act) [2] and the Surrogacy (Regulation) Act, 2021 (SR Act). Both laws came into force on January 25, 2022. The Assisted Reproductive Technology (Regulation) Rules, 2022 and the Surrogacy (Regulation) Rules, 2022 came into force on June 7, 2022 [3].

The Acts fill the existing regulatory vacuum on infertility treatments. National and State ART and Surrogacy Boards set up under the Acts will advise the government on policy matters. The National ART and Surrogacy Board was set up in May 2022 and as of March 16, 2023, 27 states and union territories had notified the creation of State Boards. The National ART and Surrogacy Registry set up in April 2022 will maintain a database of ART treatments. Appropriate authorities will register ART banks and clinics and ensure legal compliance. As of May 14, 2023, 26 States and Union Territories had notified the setting up of appropriate authorities and 27 State and Union territories had notified Sate/Union territory Boards. [4] However as per the National ART and Surrogacy Registry till 14 May 2023 registration has been given to only 219 ART clinics from the 4446 applications, 78 ART banks from 1179 applications and 122 surrogacy clinics from 854 applications [5].

We surveyed medical professionals to understand their knowledge, attitude and perception towards the Acts and to offer an initial, snapshot assessment of their impact on the medical community. The Government has already signalled its intent and published several notifications/gazettes to clarify and amend the issues surrounding the Acts [4]. We hope that these responses will help to voice thoughts, concerns and suggestions from service providers for ART to further clarify and rationalise the laws.

## Methodology of Survey

We carried out our survey over a month in October 2022. The self-administered online survey with 30 questions was circulated to a mailing list with about 30,000 gynaecologists. The questions included a mix of multiple choice, ranking and open-ended questions. Although 1113 respondents took the survey, 478 respondents completed the survey in full and the analysis that follows is based on this data (478 responses). The questions pertained to both the Acts, but we focused primarily on the ART Act given that few clinics engaged in surrogacy. We want to dispel the bias towards sensationalised media coverage on surrogacy and bring the focus back to ART.

## Survey Results

**Fig. 1:**
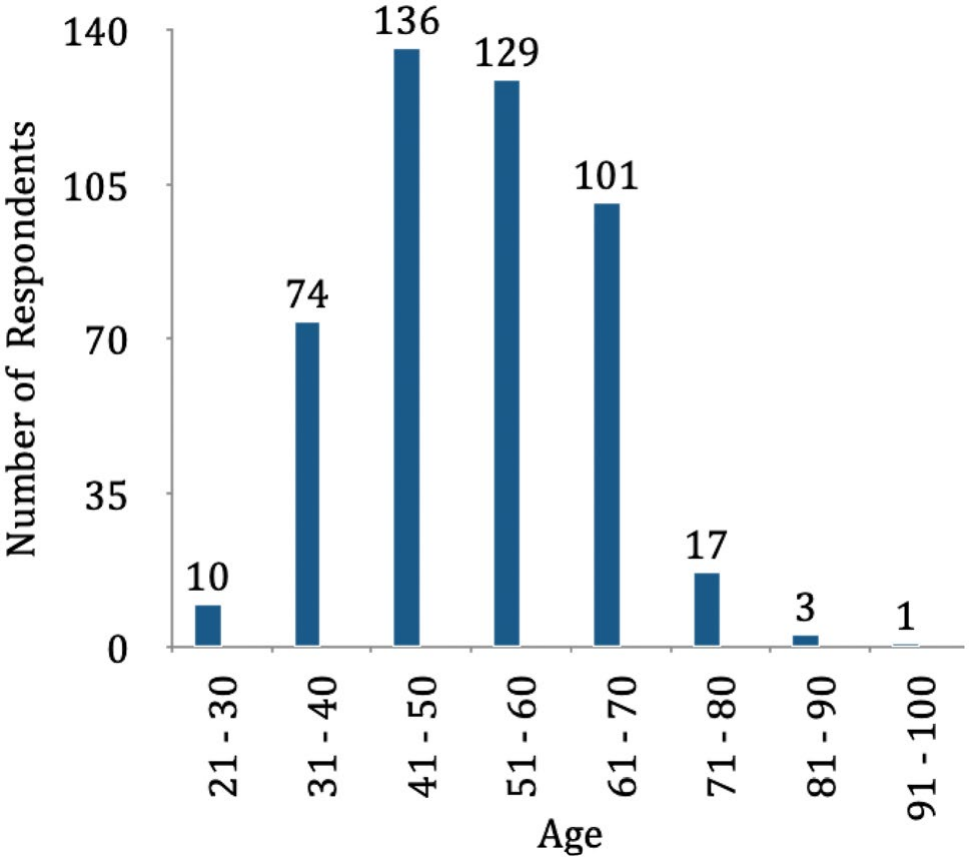
Age of respondents

The first 11 survey questions documented respondents’ profiles. Their age ranged between 26 and 91 years (Fig. 1). We had a fairly diverse geographical response with maximum responses from the West zone (Table 1). In terms of training and qualifications, 87% were obstetricians and gynaecologists while around 2% were embryologists; 2% owned ART banks and 7% responded “others”. Most of those “others” were obstetricians and gynaecologists (Table 2).

**Table 1:**
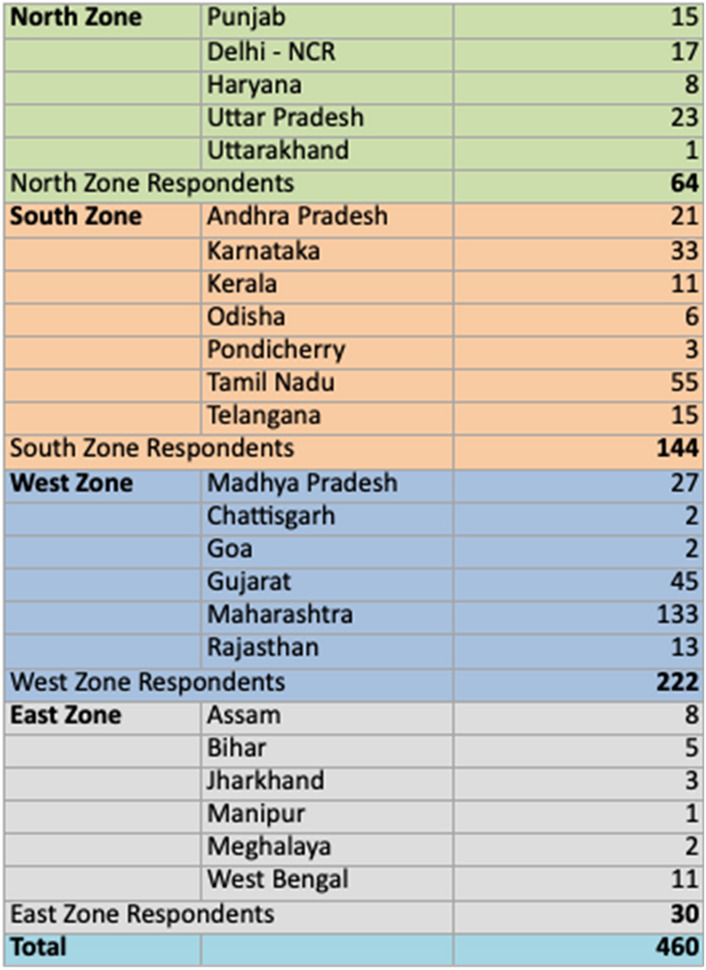
Geographical locations

**Table 2:** Speciality of respondents

Answer choices	Responses (%)	
Obstetrician and gynecologist	87.39	416
Embryologist	2.73	13
Own and manage ART bank	2.10	10
Other (please specify)	7.77	37
Total		476

**Fig. 2:**
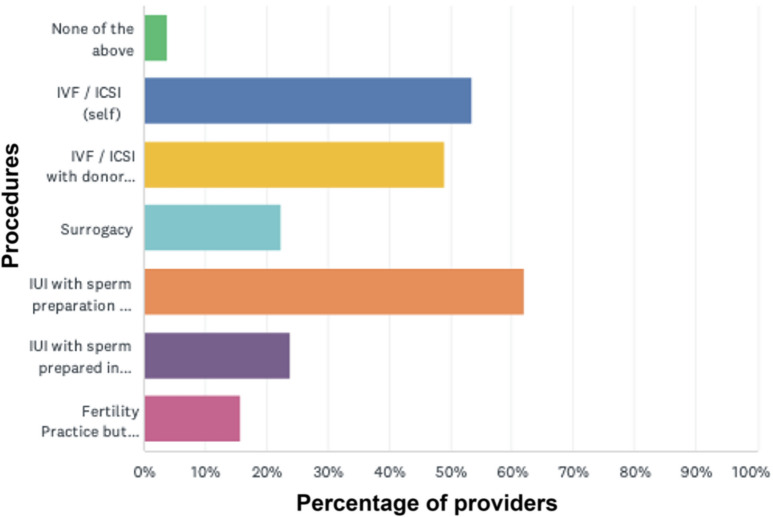
Q11 Do you perform now or were performing as a routine in the past (you can select more than one option)

As for medical procedures, only about 25% of the respondents engaged in surrogacy. The rest undertook in-vitro fertilization (IVF)/intracytoplasmic sperm injection (ICSI) with self and donor gametes and intrauterine insemination (IUI) with sperm prepared in their own or a different clinic. (Fig. 2; Table 3).

**Table 3:** Procedures performed

Answer choices	Responses (%)	
None of the above	3.79	18
IVF/ICSI (self)	53.47	254
IVF/ICSI with donor sperms/oocytes*/*embryos	49.05	233
Surrogacy	22.32	106
IUI with sperm preparation in my clinic	62.11	295
IUI with sperm prepared in another clinic	23.79	113
Fertility practice but none of the above	15.79	75
Total respondents: 475		

## Knowledge of the ART Act

We then asked about respondents’ source of knowledge of the Acts, how well they thought they knew the Acts, if they thought that the Acts were required, if they thought that the Acts were clear and what they thought the purpose of the Acts was.

When asked about their primary source of information, about 50% of the respondents said that they had read the Acts, rules and notifications. A large number also relied on webinars, lectures and CMEs (44%) and a smaller number on mainstream media (14%) (Table 4). About 54% indicated that they were moderately knowledgeable about the Acts and about 28% said that they knew a lot or a great deal about the Acts. Only 16% said they knew little or nothing at all about the Acts. (Table 5).

**Table 4:** Source of information about the acts

Answer choices	Responses (%)	
I read the acts, notifications and rules	52.52	250
Mainstream media—newspapers and television	14.71	70
Social Media—whatsapp forwards, youtube videos	24.79	118
Webinars, lectures, CME’s	44.96	214
All of the above	41.60	198
Other (please specify)	1.89	9
Total respondents: 476

**Table 5:** Knowledge of the acts

Answer choices	Responses (%)	
A great deal	11.16	53
A lot	17.89	85
A moderate amount	54.32	258
A little	15.58	74
None at all	1.05	5
Total		475

**Fig. 3:**
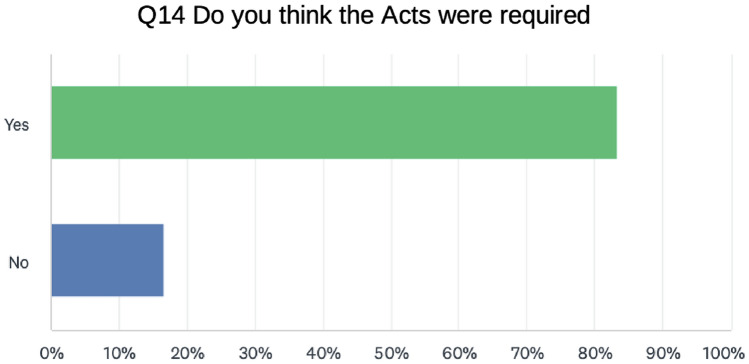
Need for the acts

Regarding the *need for the Acts*, 83% said the laws were essential while 16.74% said no (Fig. 3). Thus, medical professionals are not averse to regulation. This may reflect a generational effect as younger doctors are more accustomed to regulation according to our data.

In response to whether they had adequate clarity about the Acts, 62% thought that the provisions were not so clear or were not clear at all while 27.5% thought the provisions were somewhat clear. Only 8% thought that the provisions were extremely clear or very clear. Thus although a majority of the respondents felt that they were moderately knowledgeable about the law, there remains lack of clarity. (Table 6).

**Table 6:** Clarity on the acts

Answer choices	Responses (%)	
Extremely clear	1.68	8
Very clear	7.79	37
Somewhat clear	27.58	131
Not so clear	49.26	234
Not at all clear	13.68	65
Total		475

Contrary to perception, the medical community has welcomed the passage of the Acts. The heterogeneity in the source, extent of knowledge and clarity on the Acts underscores the need for a national effort by all stakeholders including professional associations to educate physicians, authorities and the judiciary alike on the nuances of the Acts.

## Purpose of the ART Act

On the purpose of the ART Act, respondents could tick more than one answer. The overwhelmingly large majority (86.50%) believed that it was to regulate ART through registration and specification of basic infrastructure and qualifications for personnel. Almost 40% thought that it was to impose strict punishment on medical personnel. These were followed by 19–25% who thought that it was to make ARTs accessible and affordable for all. A third thought it was to protect the rights of commissioning parents and create a fair and equitable system that is responsive to dynamic social needs; 45% thought it was to protect the rights of gamete donors and around 42% felt that it was to generate data on ART usage. Within the small number of respondents (= 32) who indicated “other”, there were concerns about corruption, creating a licence raj, a shadow ban on third party reproduction/surrogacy, undermining small IVF centres in small towns and rural areas and shoring up the interests of some doctors. The general consensus was that it needed to be amended.

## Registration Process Under the ART Act

We then focused on the registration process. Almost 45% of the respondents had registered as an ART clinic under the ART Act. Only one clinic had registered solely as a surrogacy clinic and 12% registered as both an ART clinic and surrogacy clinic. About 7.73% registered as an ART clinic and bank suggesting that very few entities chose to undertake both functions. Less than one per cent of the respondents registered as an ART Bank which is understandable given that doctors were the survey target group. (Table 7).

**Table 7:** Experience of the registration process

Answer choices	Responses (%)	
Yes—as an ART Clinic for IVF (level 2 clinic)	34.55	161
Yes as an ART Clinic for IUI (level 1 clinic)	10.30	48
Yes—as a surrogacy clinic	0.21	1
Yes—both as an ART clinic for IVF and a surrogacy clinic	12.02	56
Yes Both as an ART clinic and an ART bank	7.73	36
Yes As An ART Bank	0.86	4
No—I do not perform any procedure which needs me to register	21.89	102
No—I will stop doing procedures which need me to register under these acts	12.45	58
Total		466

Interestingly, 12% claimed that they would stop doing the procedures requiring registration. This could reflect a fear of the unknown due to a lack of clarity or misinformation about the Acts. The fear of high sentences for contravention of the ART Act is thus palpable. More knowledge dissemination and clarity on the Act is essential.

When asked if their states had set up an appropriate authority, 56% said yes while the others either said no or were not aware. Regarding the ART and Surrogacy board, 27% said that their states had a board while an equal number said not and 45% were not aware about the existence of a board. Such information must be made easily available to medical professionals.

The survey probed when registration was undertaken. Although it was spread out between April and September 2022, the highest percentage of registrations were recorded in June 2022. Thus clinics did not rush to register when the rules became public in May 2022 but waited till their notification in June 2022 to see how the process would pan out. Regarding payment, 48% were able to make the payment while 51% could not pay the registration fees. Of the 144 respondents who responded in the negative, 97 claimed that either an appropriate authority or board had not been set up or that there was no clarity on where the amount was to be paid. Another 16 claimed that the registration fees were too steep and were unaffordable as they were a small clinic or a nursing home.

On inspections, 85% of about 361 respondents claimed that they had not been visited while 15% had been inspected. Similarly 91% of the 358 clinics in the survey had not received their registration certificate, whereas 8% had received it. On the ease of registration, for 45% of the 338 clinics that responded, the process was very easy or easy; for 36% it was neither easy nor difficult and for 18% it was difficult or very difficult. Thus the National Registry as of May15, 2023,^1^ indicates that 4448 clinics, 1179 banks and 854 surrogacy clinics have filed for registration. Regarding registration fees, of the 381 clinics which responded, 66% said that the fees were too high; 12% said they were reasonable and 21% said there should no registration fees.

## Substantive Provisions of the ART Act

We then queried the adequacy and reasonableness of the ART Act’s substantive provisions on minimum conditions for an ART clinic, the duties and obligations of ART clinics, the age limits for commissioning couples, the period of proven infertility, protection for gamete donors, and provisions on gamete transfer, pre-implantation genetic testing and offences.

Regarding qualifications for clinic personnel, about 55% agreed that they were adequate, 13% neither agreed nor disagreed and 23% disagreed. Almost 70% agreed on the list of minimum equipment required under the ART Act.

Regarding the clarity and accessibility of forms in the ART Act rules, 45% agreed that they were clear and accessible, 18% neither agreed or disagreed and 26% disagreed. A minority of 10% were unable to comment. On the feasibility of setting up grievance cells, 29% felt they were feasible, 18% neither agreed nor disagreed and almost 40% disagreed that they were feasible. Another 12% had no view on this. On duties and obligations imposed on the ART clinics, 39% found them reasonable, 17% neither agreed nor disagreed, 34% found them unreasonable and almost 9% had no view.

A large percentage of the respondents (74%) viewed the age limits set on the commissioning parents as reasonable and 15% found them to be unreasonable. For the period of one year of unprotected coitus before treatment is started, 57% were in agreement and 24% thought it was not acceptable. Again 50% of the respondents thought that the protection for egg donors was adequate while 24% disagreed; 9% had no view and 16% neither agreed nor disagreed.

On the role of ART Banks in recruiting and screening donors with the clinics performing the oocyte retrieval, 44% thought that the provisions were clear while 12% neither agreed nor disagreed and 30% felt that the previsions were unclear. Asked if Section 29 which prohibits the sale or transfer of gametes or embryos unless it pertains to one’s own gametes was reasonable; 45% thought it was reasonable; 30% thought it was unreasonable and 14% neither agreed not disagreed. On the adequacy of PGD testing, almost 45% agreed, 20% disagreed and 21% neither agreed nor disagreed. Regarding offences and penalties, a majority of respondents (53%) viewed the minimum mandatory sentences under Sections 32 and 33 of the ART Act as unreasonable while 18% thought they were reasonable and 13% neither agreed not disagreed. Regarding Section 34 which punishes any contravention of the Act for which there is no punishment with a stringent fine (5–10 lakhs) followed by imprisonment for 3–8 years for second contraventions, 47% disagreed that it was reasonable; 15% agreed that it was reasonable and 19% neither agreed nor disagreed.

## Donor Programs

Given the ART Act’s stringent provisions on gamete donation and use, we queried its impact on donor programs, including the scientific feasibility of having an Ovarian Hyperstimulation Syndrome (OHSS)-free program, the strict limits on gamete donation and sharing, accessing ARTs and finally the likely impact of the ART Act on the availability and affordability of gametes.

70% of the respondents believed that OHSS-free donor programs were possible, signalling scientific advancements in the safe retrieval of oocytes. On the feasibility of stimulating and retrieving 7 mature oocytes, 76% of the respondents disagreed; only 13% agreed and only 5% neither agreed nor disagreed. On the rule of egg donation once in a lifetime, around 70% of the respondents thought that it was unreasonable; 15% felt that it was reasonable and 10% neither agreed nor disagreed.

A similar profile of responses emerged on sperm donation being restricted to only one commissioning couple. 74% thought it was unreasonable while 15% thought that it was reasonable and 6% neither agreed nor disagreed.

Regarding clarity about insurance products for donors, 56% disagreed, 15% agreed and 15% neither agreed nor disagreed.

On whether the ART Act adequately protected egg donors, 39% agreed, 31% disagreed and 18% neither agreed nor disagreed. A majority of respondents viewed the requirement for the commissioning couple to obtain affidavits from a Metropolitan Magistrate or Judicial Magistrate as unreasonable (62%); 19% thought it was reasonable and 10% neither agreed nor disagreed. *A notification was issued on October 10, 2022 which amended this requirement to include a notary public.*

On whether donor programs will remain affordable, 75% disagreed, 12% agreed and 7% neither agreed nor disagreed. On whether commissioning couples would have certainty as to the availability of gametes, 61% disagreed, 13% agreed and 15% neither agreed nor disagreed. On the adequate availability of sperm and egg donors, 75% disagreed, 10% agreed and 7% neither agreed nor disagreed.

The medical community is most disappointed with the gamete donation provisions of the ART Act. In India, reportedly 35–40% of all IVF cycles are donor cycles because IVF is expensive and involves out of pocket expenditure, hence couples wait until they are older to access ARTs which produces a diminished result. In rural areas TB is a major cause of infertility, whereas in urban areas, endometriosis affects and destroys the ovaries. Doctors report that donor cycles in their clinics have stopped as of March 2022 since most rely on an ART bank and cannot access donor gametes pending the Bank’s registration. This delay and the shortage in gametes will likely increase costs of fertility treatment (conservatively speaking) by 125%. Doctors thus believe that the law is effectively abandoning infertile couples who cannot afford the high price of treatment.

## Revisiting Whether Act Achieves its Objectives

We asked respondents whether the ART Act achieved its objectives. We posed these questions earlier in the survey and repeated them to assess any differences once respondents had thought through the ART Act’s provisions. Predictably, only 60% now (as opposed to 86% earlier) thought that the ART Act’s goal was to register clinics and banks and specify minimum physical and medical infrastructure. A higher percentage (55% as opposed to 40% earlier) thought that the ART Act aimed to punish medical personnel. 49% disagreed that the ART Act sought to be gender inclusive but 32% agreed that the Act empowered family formation by all genders. This is higher than the 19% of respondents who at the start felt that the ART Act aimed to be gender inclusive. On the accessibility and affordability of ARTs, 69% disagreed that the ART Act sought to make ARTs accessible for all and 71% disagreed that it aimed to make ARTs affordable for all. Less respondents now thought that the Act’s purpose was to make ARTs accessible (from 28 to 20%) and affordable (from 24 to 18%). More respondents thought that the Act aimed to protect commissioning couples (from 33 to 40%). The number who thought that it protected gamete donors remained roughly the same. A much higher number of respondents now thought that the Act sought to generate data on ART usage (from 42 to 75%). Slightly more respondents disagreed that the Act was aimed at creating a fair and equitable system that was responsive to social needs (35% over 33%). Almost 27% thought that none of the Acts’ listed aims matched their purpose. Of the 5 respondents who indicated “Other”, one respondent said the ART Act “increases the stress of the ivf clinicians effectively”; “is…against the basic reproductive right of the couples”, will increase social disparity, will tie down couples in bureaucratic hurdles, and should instead make ARTs “affordable and accessible to all, doctors, patients & donors.”

## Respondents’ Views on the ART Act

Finally we invited respondents to express their views on the Acts. Of the 128 respondents who did so, all barring 3 expressed concern with the Acts. These included the high registration fees, scope for corruption arising from regulation, high levels of punishment, the push to corporatize IVF practice and the urgent need for amendments. Only 5 doctors expressed concern with the law’s impact on surrogacy. Respondents’ most significant concern pertained to the law’s effect on donor cycles (35) and the affordability of IVF (24). We address each issue in turn.

Doctors, like all professionals displayed scepticism of the draconian provisions. They worried that hyper-regulation of the medical profession would lead to unwarranted interventions that would benefit state officials and lawyers. Here their experience with the implementation of the PCPNDT Act loomed large. One respondent noted that the “interpretation of the act is left on to local authorities and that is going to cause lot of harassment as in PC- PNDT. It would have been better if it was controlled and interpreted by a central authority like ICMR along with a fair representation of national societies to balance the fair implementation of the act.”

Medical professionals’ fear was intensified by high penalties (including minimum mandatory penalties) under the Acts and their strong sense that infertility doctors are viewed as culprits.

Several respondents complained about high registration fees for ART clinics and banks^2^ on top of other regulatory requirements which were onerous for small nursing homes and hospitals.

These fees would make fertility practice in smaller tier 2 and 3 towns unfeasible. This fee structure would shut down small, private providers of fertility services necessitating large scale investment in government fertility services. One respondent suggested that the government take steps to “initiate these services in government hospitals rather than putting pressure on private practitioners who are the backbone of our strong health system in India.”

Respondents viewed the laws’ mandates on donor programs as the most problematic. The ART Act’s requirement that gamete donation not be compensated, that a donor’s gametes (sperm and oocytes) are used only by one couple and the limitations on the number of eggs and number of times that an egg donor can donate eggs would lead to a shortage of gametes. Gamete shortage would increase the costs of IVF cycles making it unaffordable for couples. Further, “poor patients won't be even able to have IUI with donor samples due to increased cost.” “ART will become very expensive for patients of low socioeconomic backgrounds”. Restrictions on egg donation would increase the costs of a donor cycle threefold. Respondents opined that that it was unrealistic to expect third party egg donors or even relatives to undergo the inconvenience of a surgical procedure for no compensation, which would reduce the number of donor cycles.

Another respondent noted the social impact of the ART Act.

The clause of a judicial magistrate’s signature for the donor egg programme is changed to a Notary Public. That is better but should be done away with. 7 oocytes only is impractical. Not allowing sharing of eggs among two recipients and restricting a donor to donate only once in her life makes donor egg cycles too expensive. By stopping commercial surrogacy, there will be more illegal transactions exposing surrogates to greater danger. Having a separate counsellor and grievance cell is superfluous for stand-alone single doctor run ART clinics. Severe penalties to doctors are unfair. Inadvertent mistakes should be let off with a warning. Donating sperms by a sperm donor only once is also highly unreasonable and will make costs exorbitant for an IUI with donor sperm Fertility is an important part of society. Raising costs and making treatment unaffordable may cause break up of marriages and more pain in society. Rules related to fertility treatment hence cannot be so restrictive.

A few respondents noted that the SRA was full of ambiguities and contradictions, and that women would not undertake altruistic surrogacy.

## Conclusion

As our survey shows, the passage of the Acts has been welcomed by health care providers as it fills a regulatory vacuum. Although a majority of respondents think that they have a good understanding of the new laws, there is considerable uncertainty on the ground. Doctors desire regulation but are ambivalent whether the Acts satisfy their stated aims and objectives. They find various provisions onerous. Far from assisting infertile couples, particularly those requiring third party reproduction, the laws will in fact deny them the benefits of ARTs. Those who manage to form their families will likely do so at great cost and over long periods of time. This is reflected in the fact that more than a year since the ART Act and SRA became law, several challenges have made their way in the High Courts and the Supreme Court.

If reform were possible, our respondents suggest reducing the costs of registration, relaxing the qualification threshold for a new doctor setting up an ART clinic and relaxing qualifications for a level 1 IUI clinic. They also suggest reducing punishments against doctors. Since both Acts impose prison sentences and heavy penalties, some clinics will likely leave the ART sector because of the risk of prosecution. Attempts to criminalise medical practices have been hard to enforce in the past. Proposals that seek to make infertility care “accessible, acceptable and affordable” suggest permitting increased donation while safeguarding the interests of donors (allowing egg donors to donate up to 3–6 times with compensation under a central registry like COWIN portal), removing the requirement for an egg donor to have her husband’s consent, removing the need for an affidavit to be signed by a judge (now amended), giving the commissioning couple the choice between frozen and fresh gametes, increasing the insurance coverage for egg donors to 5 lakhs, allowing sperm samples to be used for 5–6 couples, and increasing the maximum age for the surrogate to 45 years from 35 years as she may not have completed her family by 35.

The ART Act should protect the donor’s right to counselling and to withdraw her consent while compensating her for medical expenses, lost time, lost wages, and inconvenience caused by treatment and an egg retrieval procedure. Surrogates similarly need compensation for lost wages, ten months of reproductive labour in pregnancy, for lost services to their families, and for giving birth. As is evident in our survey, the ART Act demands that more gamete donors step forward while simultaneously disincentivising them. Alongside gamete shortage, the SRA criminalises any intermediation by agents, brokers, or clinics to locate a surrogate so commissioning parties have to find an altruistic surrogate on their own, adding to the time and expense involved. Couples will in effect turn to close relatives/friends to undertake surrogacy. The sharp edge of the knife of this Act will fall on economically vulnerable egg donors and surrogates who occupy the pedestal of altruism with no protection. The Acts may contribute to the development of an illegal, informal market in egg donation and surrogacy services. The cost and effort to undergo ART and surrogacy will only increase exponentially.

As they stand today the ART Act and SRA will cause both sectors to likely shrink substantially. This will severely compromise access in a space where access is already limited. The fact that the registry has received 4446 applications for ART clinics and only 854 applications for surrogacy clinics reflects the reduction in providers willing and able to provide surrogacy services [5].

Infertility is already a much stigmatised problem which deserves to be a higher public health priority. While the laws are welcome, clarification and changes in both laws is the need of the hour to make ART more accessible, available and affordable to the millions of couples who need these services and the health care providers who deliver them.

## Data Availability

Data and material will be available by request.

## Footnotes

1 https://registry.artsurrogacy.gov.in/

2 The fee structure under the ART rules are as follows; Rs 50,000 for Level 1 ART clinic; Rs 2,00,000 for Level 2 ART clinic; and Rs 50,000 for ART bank.

## Code Availability

Software application or custom code.
